# Enrichment of Starch Desserts with the Addition of Apple Juice and Buckwheat Fiber

**DOI:** 10.3390/polym15030717

**Published:** 2023-01-31

**Authors:** Greta Adamczyk, Paweł Hanus, Inna Bobel, Magdalena Krystyjan

**Affiliations:** 1 Department of Food Technology and Human Nutrition, Institute of Food Technology and Nutrition, University of Rzeszow, 4 Zelwerowicza St., 35-601 Rzeszow, Poland; 2 Department of Bakery and Confectionary Goods Technologies, Educational and Scientific Institute of Food Technology, National University of Food Technologies, 68 Volodymyrska St., 01601 Kyiv, Ukraine; 3 Department of Carbohydrate Technology and Cereal Processing, Faculty of Food Technology, University of Agriculture in Krakow, Al. Mickiewicza 21, 31-120 Krakow, Poland

**Keywords:** potato and corn starch gels, buckwheat fiber, apple juice, pasting characteristic, texture parameters, polyphenol content

## Abstract

Buckwheat hulls which are rich in fiber are good ingredients to increase the nutritional value of food products. The aim of this study was to determine the effect of the applied additives in the form of fiber and apple juice on the properties of both potato and corn starch (normal and waxy). In order to characterize the rheological properties of kissel, the pasting characteristic was measured. In the obtained gels, the basic quality parameters were determined. The analysis of texture, color parameters, and also total polyphenol content were determined. Buckwheat hulls, in addition to their high fiber content, are a valuable source of phenolic compounds and can be a great additive in starch desserts. The addition of buckwheat hulls and apple juice improved the nutritional value of the final products but also caused changes in the technological qualities: it increased the initial temperature of potato starch mixtures (by approx. 9 °C); it decreased the viscosity of cold desserts (from 8 to 55%); and increased the hardness of the final product by more than 7 times. In the case of other starches, the recorded changes were much smaller than for potato starch-based products.

## 1. Introduction

Starch is a reserve material of many plants, and from a chemical point of view, it is a semi-crystalline polysaccharide composed exclusively of D-glucose monomers connected by α-glycosidic bonds. Starch granules contain two fractions of polysaccharide chains: unbranched amylose (AM) and branched amylopectin (AMP). AM is composed of relatively long, linear anhydroglucose chains connected in about 99% by α-(1,4)-glycosidic bonds and in a maximum of 1% by branching α-(1,6)-glycosidic bonds. In turn, the branched structure of AMP results from a much larger amount of α-(1,6)-glycosidic bonds, which is estimated at approximately 5% [1]. AM usually constitutes from 10 to 35% of starch weight, which depends on its botanical origin [2]. The exception, however, are so-called waxy starches, which consist solely of amylopectin [3]. Currently commercially available are waxy starches obtained by a natural selection of varieties derived from potatoes, maize, and rice [4]. In addition, natural mutants have been identified among other plant species that contain waxy starch, e.g., barley, sorghum, and amaranth [5,6]. Since waxy starches do not contain amylose, they are characterized by unique functional properties [7,8]. Starches are widely used as stabilizing, thickening, or gelling and bulking agents in the formulation of food products [9,10]. Moreover, starches are base materials for the formation of biodegradable packaging films and edible coatings [11,12,13,14,15]. The botanical origin of starch determines its properties and therefore affects the quality of starch-based food products, mainly their texture and rheology [16].

Desserts are a wide assortment of sweet food products which include jellies, kissels, puddings, creams, mousses, and ice creams, as well as confectionery products. These foods can be served both cold and hot [17]. Kissel is a starch-based dessert prepared using water, powdered juice or juice concentrate, and the puree of cooked fruit. Potato starch or oxidized starch are most commonly used as gelling agents in the production of kissel. An alternative to chemically modified starches can also be native starches of waxy varieties, which are characterized by favorable rheological properties and appropriate transparency [18,19,20]. However, due to its high carbohydrate content, kissel may seem to be a highly caloric food product. With the introduction of various ingredients, these foods can be enriched with many valuable nutrients and bioactive substances. A key issue in the development of functional foods is the production of food enriched with dietary fiber [21,22,23]. The recommended daily dietary fibre intake for an adult should be about 25–35 g, of which about 50–75% should be of the insoluble fraction (IDF) [23,24]. The enrichment of nutrient-poor foods allows us to expand and diversify our diets, enables us to obtain valuable nutrients from a variety of food sources, and reduces the risk of deficiency of valuable micro- and macroelements. Food fortification has grown strongly in recent years [25,26,27,28]. The latest studies indicate that it is possible to incorporate dietary fiber into many products. Canalis et al. (2019) [25] showed that fiber and inulin incorporation changed the water loss profile and the rate for dough. Niño-Medina (2019) [29] used dietary fiber extracted from soybean and chickpea husks in the formulation of white bread. Other authors proved that healthier low-fat Italian-type salami may be produced with inulin or fructooligosaccharides as a substitute for fat from pork with good technological and sensorial parameters [30]. Dietary fiber from outer leaf powder of Chinese cabbage (a by-product of kimchi) can be used positively used to improve muffin quality, texture properties, and sensory evaluation [31]. Beltrán-Medina (2020) [32] used a coffee industry by-product in a cereal-based extruded food product.

A specific form of raw material that is used is raw by-products, which in the aspect of one industry is a production residue, but in another is a valuable source to be reused in an efficient manner. By-products rich in fiber are important as health-promoting components. Buckwheat hulls are a good ingredient to increase the nutritional value of food products. They have more bioactive compounds than buckwheat flour. Buckwheat hulls were used as an additive in products as a component to noodles or yogurt [33,34].

The effect of the addition of buckwheat hulls on the rheological properties of gels based on normal and waxy potato starch was studied in our previous work [35]. In those samples, buckwheat hulls were used in the amount of 0.2%. In our current work, we decided to use a smaller amount of buckwheat fiber (0.05% and 0.10%) in systems with apple juice, which gave the gels both color and a sweet taste. The aim of the study was to determine the effect of the applied additives—fiber and apple juice—on the properties of potato and corn starch and to use such additives to enrich sweet starch-based desserts with dietary fiber and apple juice. Such a procedure will increase the nutritional value of products that consumers usually associate with empty calories. In addition, it will enable the use of the post-production product and indicate its application possibilities.

## 2. Materials and Methods

### 2.1. Materials

The experimental material consisted of: normal potato starch (PS) (Superior Standard, Wielkopolskie Przedsiębiorstwo Przemysłu Ziemniaczanego S.A., Lubon, Poland), waxy potato starch (WPS) (Eliane 100, AVEBE FOOD, Veendam, The Netherlands), normal corn starch (CS) (Cargill, Schiphol, The Netherlands), waxy corn starch (WCS) (Cargill, Soborg, Denmark), organic buckwheat fiber (BH) (Look Food Sp. z o. o., Warsaw, Poland), and concentrated apple juice (CAJ) (APKON, Przemysl, Poland). The apple juice (AJ) was prepared by diluting the concentrate with water in a ratio of 1:1. Buckwheat fiber (BH) contains 100% of the ground roasted buckwheat hulls from controlled organic farming and has the following parameters: 4.5% protein, 3.1% carbohydrates, and 0.6% fat.

### 2.2. Methods

#### 2.2.1. Material Characterization

Dry matter: Determination of the dry matter content in the preparation of starch and buckwheat fiber was carried out in a dryer (Binder, Tuttlingen, Germany). This method consists of the evaporation of water from the product sample in the process of drying at 130 °C for 60 min and determining the residual dry matter by weight.

Fiber content: According to AOAC (2006) [36], the fiber content of the buckwheat hulls were determined.

Brix value: Apple juice solution added to the examined samples (apple juice diluted with water 1:1) was established using a PAL-1 refractometer (Atago, Fukuoka, Japan) with a Brix scale (%). The results were averaged, and the standard deviation was calculated.

pH: The pH of the prepared apple juice solution was measured using a Crison Instruments S.A. pH-meter (Barcelona, Spain).

#### 2.2.2. The Fruit Gels (Kissel) Preparations

Fruit gels (kissel) were produced under laboratory conditions. The ingredients used in the production of kissel are listed in Table 1. To produce the kissel, all the ingredients were mixed in an appropriate amount of water. The samples were heated up to 95 °C, kept for 5 min at this temperature, and then cooled down to 50 °C.

#### 2.2.3. Pasting Characteristics of Kissel

The pasting characteristics of starch suspensions were carried out using a Brabender viscograph (Brabander^®^ GmbH & Co. KG, Duisburg, Germany) with the procedure according to Adamczyk et al. (2020) [37]. The aqueous suspensions of starch blends were prepared at ambient temperature, and pasting behavior was measured for 83 min. Pasting characteristics of the samples were run with the following parameters: heating from 30 to 95 °C with 1.5 °C/min, maintaining samples for 5 min at 95 °C, and cooling from 95 to 50 °C with 1.5 °C/min at constant agitation of 75 rpm. Measurements were performed in duplicate.

#### 2.2.4. Texture Parameters of Kissel

Texture parameters of kissel were established using the Universal Testing Machine EZ-Test EZ-LX (Shimadzu, Kyoto, Japan), equipped with an aluminum probe (d = 7 mm, h = 40 mm), at a force of 20 N. Compression was performed until 50% of the sample height at a speed of 50 mm/min. Measurements were made in 4 replications. The hardness of the obtained samples [N] was determined as one of the texture parameters [37].

#### 2.2.5. Color Measurement of Kissel

Quantitative color measurements were conducted using an UltraScan VIS spectrophotometer (HunterLab, Reston, VA, USA) according to the CIE Lab model using diffuse/8° geometry with automatic mirroring on and off. The bandwidth in the optical system was 10 nm and the range was from 360 nm to 780 nm. The spectrophotometer recorded L*, a*, and b* coordinates defined as: lightness where 100 is white and 0 is black (L*), redness+/greenness− (a*), and yellowness+/blueness− (b*). Measurements were made in triplicate. The mean values of the measurements were calculated taking into account the standard deviations [38].

#### 2.2.6. Analysis of Total Polyphenol Content and Antioxidant Properties of Kissel

The methanol extract was prepared for analysis of the total polyphenol content and antioxidant properties. Fresh kissel samples (10 g) were treated with a 70% methanol solution (20 mL). The extraction process was carried out using an ultrasonic bath (Polsonic, Warsaw, Poland) (30 min at 25 °C). After this process, the samples were centrifuged for 10 min at 7000 rpm. The obtained supernatant (methanol extract) was used for further analysis.

The total polyphenol content in kissel was assessed using the Folin–Ciocalteu phenol reagent with the method described by Singleton and Rossi (1999) [39]. The reaction mixture contained extract (0.1 mL), distilled water (2.0 mL), Folin–Ciocalteu reagent (0.2 mL), and 20% (*w*/*v*) sodium carbonate solution (1.0 mL). The absorbance of the samples was measured at 765 nm against distilled water using a spectrophotometer (Nicolet Evolution 300, Thermo, Waltham, MA, USA). Measurements were performed in triplicate. The total polyphenol content was expressed as gallic acid equivalents (GAE) in milligrams per 100 g of kissel (GAE; mg/100 g).

According to Re et al. (1999) [40], the antioxidant activity was carried out using the ABTS^+^ cation radical. The reaction mixture consisted of adding the sample (0.03 mL) and the ABTS radical solution to the water (3.0 mL). The absorbance at 734 nm, against distilled water, was measured after 6 min of reaction using a spectrophotometer (Nicolet Evolution 300, Thermo, Waltham, MA, USA). Measurements were performed in triplicate.

The scavenging activity was measured according to the elimination of DPPH (1,1-diphenyl-2-picrylhydrazyl) free radicals [41]. The reaction mixture consisted of adding the sample (0.5 mL) and the DPPH radical solution in methanol (2.0 mL). The absorbance at 517 nm, against methanol, was measured after 10 min of reaction using a spectrophotometer (Nicolet Evolution 300, Thermo, Waltham, MA, USA). Measurements were performed in triplicate. The values of the antioxidant activity (ABTS, DPPH) of studied samples were expressed in mM TE/100 g kissel (Trolox Equivalent).

#### 2.2.7. Statistical Analysis

Statistical analysis of the results was performed using Statistica v.13.3 (StatSoft, Inc., Tulsa, OK, USA). For all determinations, one-way ANOVA was carried out using Duncan’s test at a confidence level of α = 0.05.

## 3. Results

### 3.1. Material Characterization

The dry matter content of analyzed starches were in range 82.32–89.22% and buckwheat fiber was at the level of 89.40%. The prepared apple juice (AJ) used in formulation showed the following parameters: pH 3.45 and 36.10% Brix. The buckwheat hulls had 71.40 g/100 g total dietary fiber (TDF) and included insoluble dietary fiber (IDF) 62.06% and soluble dietary fiber (SDF) 11.55%.

### 3.2. Determination of the Pasting Characteristics

**Figure 1:**
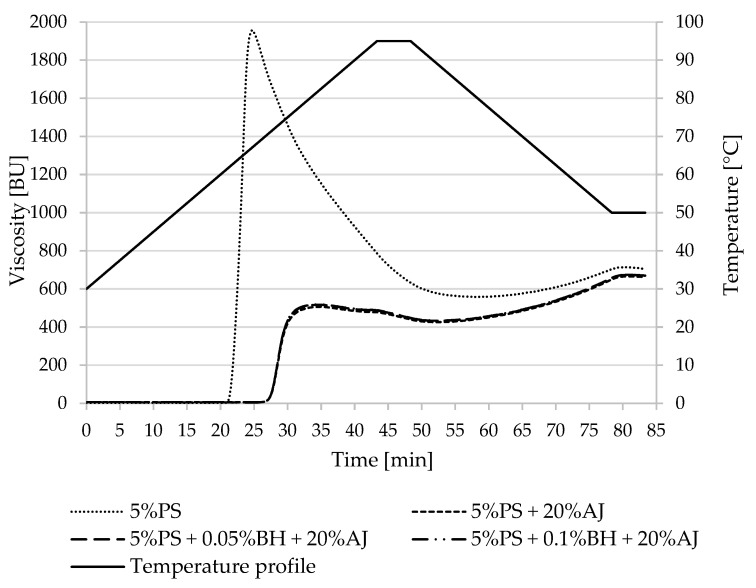
Pasting characteristics of 5% PS and its mixture with apple juice and buckwheat fiber. PS—potato starch, BH—buckwheat fiber, and AJ—apple juice.

**Figure 2:**
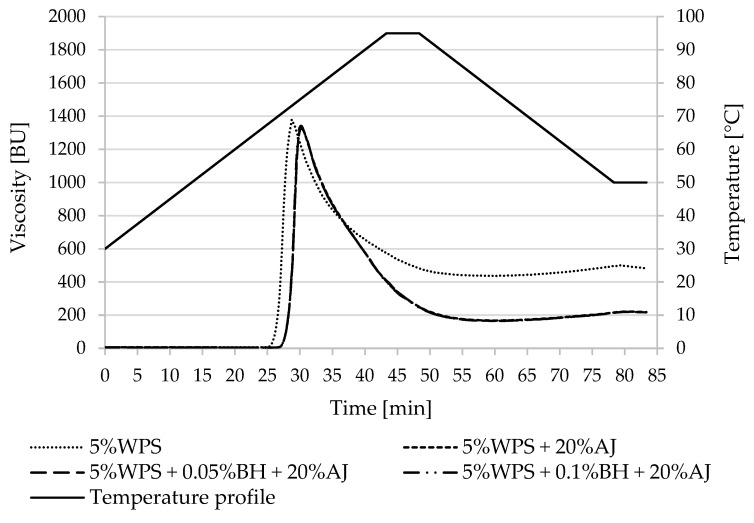
Pasting characteristics of 5% WPS and its mixture with apple juice and buckwheat fiber. WPS—waxy potato starch, BH—buckwheat fiber, and AJ—apple juice.

**Figure 3:**
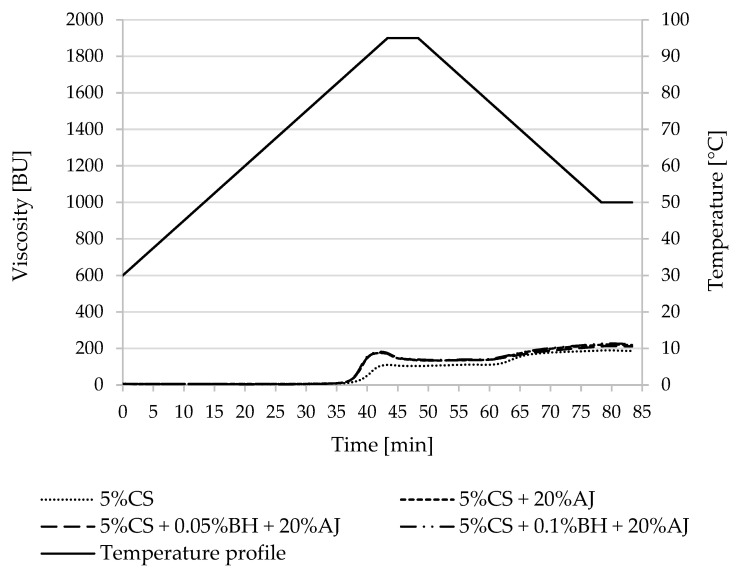
Pasting characteristics of 5% CS and its mixture with apple juice and buckwheat fiber. CS—corn starch, BH—buckwheat fiber, and AJ—apple juice.

**Figure 4:**
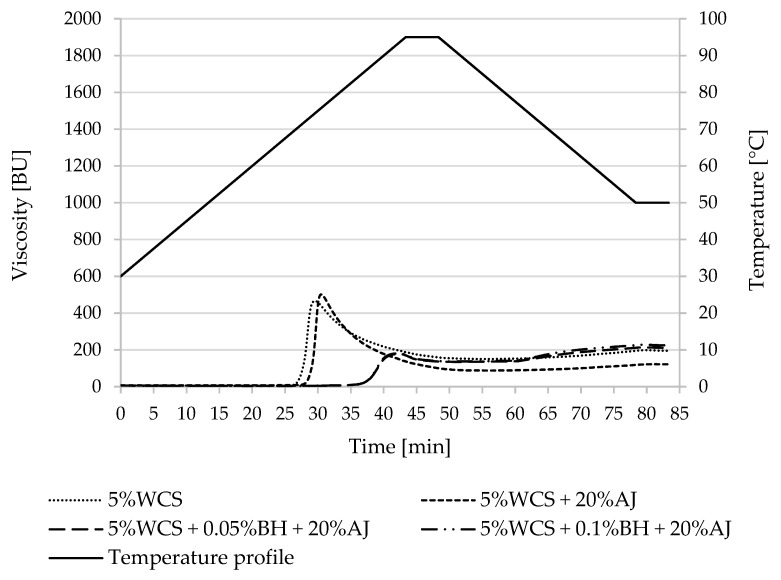
Pasting characteristics of 5% WCS and its mixture with apple juice and buckwheat fiber. WCS—waxy corn starch, BH—buckwheat fiber, and AJ—apple juice.

Pasting property parameters of potato and corn starches and the designed desserts were summarized in Table 2 and Table 3 and Figure 1, Figure 2, Figure 3 and Figure 4. The difference in the pasting properties among various starches and the obtained kissel was observed. The gelatinization characteristics of starch in the tested samples with juice and fiber (AJ and BH) were compared with the gelatinization characteristics of pure starches (5%PS, 5%WPS, 5%CS, and 5% WCS). 

The beginning of pasting (T_0_) for PS (61.2 °C) had smaller value in comparison to juice systems present (5%PS + 20%AJ, 5%PS + 0.05%BH + 20%AJ, 5%PS + 0.10%BH + 20%AJ) (Table 2). This means that the addition of juice caused a delay in the pasting as the T_0_ parameter was a higher temperature (70.1 °C) than in the starch without additives (61.2 °C). Regardless of whether buckwheat fiber was present in the system with normal potato starch with juice or not, its effect on this parameter was not observed. A similar dependence of the T_0_ parameter was noted in the samples with WPS. Consequently, the presence of apple juice increased the temperature of the beginning of pasting from 67.6 °C (WPS) to 70.0–70.1 °C.

The studied normal potato starch samples reached a higher value of peak viscosity (η_max_) (1957.0 BU) in comparison with waxy starch (1387.0 BU).

The maximum viscosity (η_max_) of PS (1957.0 BU) decreased in systems with apple juice by about 75% (507.0–518.5 BU) and by about 4% in the WPS samples (from 1387.0 BU to 1328.0 BU). The higher temperature at maximum viscosity (T_ŋ max_) was noted in PS with juice and fiber (80.7–81.4 °C), which was higher than PS (66.7 °C). The same effect was observed in the case of waxy potato starch but differences between temperatures were lower, about 3 °C (72.9 °C for WPS, and 75.0 °C for samples with AJ and BH).

During the heating of samples to 95 °C, the viscosity was decreasing. In the PS sample, this value decreased from 1957.0 BU to 787.0 BU (about 1200 BU), and in the PS samples with AJ and BH, from 518.5–507.0 BU to 477.5–488.5 BU (about 30.0 BU). In the waxy potato starch, viscosity decrease was similar in the control sample (from 1387.0 BU to 575.0 BU) and the waxy starch with juice and fiber (from 1328.5–1341.0 BU to 400.5–407.5 BU).

The highest breakdown parameter was observed in the PS sample (1325.0 BU), and this value was significantly reduced when the juice was present in the normal potato starch system (66.5–70.5 BU). The opposite effect was noted in the waxy potato starch system. The addition of apple juice caused an increase in the BD parameter of the WPS (from 904.0 BU to 1079.0–1094.5 BU).

Setback values were higher for the normal potato starch system than for waxy starch. The addition of apple juice caused an increase of setback parameters in PS, from 70.5 BU to 209.5–211.5 BU, and in WPS, from 7.5. BU to 32.5–34.5 BU.

The normal potato starch with juice showed lower final viscosity values (649.5–658.0 BU) compared to the control sample (706.0 BU).

The temperature of the beginning of pasting of normal corn starch was different than in the case of normal potato starch. Namely, the addition of buckwheat hulls and juice caused a decrease in this parameter, not an increase. A decrease in this temperature, along with the addition of juice and fiber, was observed in waxy corn starch. In both cases, normal and waxy corn starch had a higher peak viscosity when compared with the control starch samples (CS and WCS).

The final viscosity of normal corn starch (CS) was different than in natural potato starch (PS) because, in this case, the control sample (CS) had a lower value of viscosity at 50 °C (191.5 BU) than corn starch combined with juice and fiber (211.0–224.0 BU). On the other hand, the final viscosity (η_50 °C_) of corn starch and starch with additives had higher values than peak viscosity values (η_max_), as opposed to that in normal and waxy potato starch.

In waxy corn starch, the presence of acidic juice contributed to the reduction of the final viscosity of the tested systems, from 196.0 B.U (WCS) to 110.0–118.5 BU (WCS + AJ and WCS + BH + AJ).

### 3.3. Texture Analysis of Kissel

The results of the texture analysis of fruit gels with buckwheat fiber are presented in Table 4 and Table 5. The same tendency was observed in case of potato (Table 4) and corn starch (Table 5) kissel gels. 

The addition of apple juice and fiber to potato and waxy potato starch changed the hardness of starch gels. The hardness of 5%PS increased from 0.044 N to 0.330 N in the sample with juice (5%PS + 20%AJ). Nevertheless, the presence of fiber in fruit gel decreased the hardness to 0.180 N (5%PS + 0.05%BH + 20%AJ) and 0.100 N (5%PS + 0.10%BH + 20%AJ). The opposite effect was observed in waxy potato starch mixtures (Table 4). The control sample (5%WPS) had the highest value of hardness (0.023 N), and other ingredients (juice and fiber) caused a decrease in this value. The waxy potato gel had the lowest value with 0.10%BH (0.018 N). On the one hand, the values of waxy gels with fiber were smaller than the control ones, but as in the case of potato starch, a higher share of fiber in the sample resulted in lower hardness value.

In the samples of corn starch gels, the effect of the addition of apple juice and fiber on the hardness of the gels was also observed (Table 5). A clear increase in this parameter was observed in the native starch gel (5% CS), where the value from 0.199 N increased to 0.380 N, 0.390 N, and 0.510 N in 5%CS + 20%AJ, 5%CS + 0.05%BH + 20%AJ, and 5%CS + 0.10%BH + 20%AJ, respectively. On the other hand, in the waxy starch gel (5%WCS), the opposite effect was observed. Namely, a clear decrease in the hardness values in the gels were 0.021 N, 0.019 N, and 0.018 N (5%WCS + 20%AJ, 5%WCS + 0.05%BH + 20%AJ, and 5%WCS + 0.10%BH + 20%AJ) compared to the control gel (0.027 N).

### 3.4. Color Measurement of Kissel

The Table 6 and Table 7 show the results of the color parameters (L*, a*, and b*) of fruit kissel prepared based on 5% (*w*/*w*) potato and waxy starches and additives such as apple juice (20%) and buckwheat fiber (0.05 and 0.10% in formulation). The color parameters were measured not only for the designed kissel, but also for the starch gels without the addition of apple juice and buckwheat hulls, in order to observe the influence of the botanical origin and type of starch on the value of L*, a*, and b* parameters.

Potato starch gels were characterized by a higher L* parameter in the case of waxy starch (76.07) than in the case of normal starch (70.72), which means that the WPS sample had a greater share of white color (Table 6). The addition of flavoring substances (apple juice and buckwheat fiber) significantly reduced the value of the parameter L* of PS gel, thus indicating a darker color of the kissel samples. However, between samples 5%PS + 20%AJ, 5%PS + 0.05%BH + 20%AJ, and 5%PS + 0.10%BH + 20%AJ there were no statistically significant differences in the values, which means that the addition of buckwheat hulls in the amounts of 0.05% and 0.10% did not affect the L* parameter, only the color of the juice used was important here.

Parameter a* indicates that samples with juice and fiber had more redness (a* > 0) compared to the starch gel (a* < 0) because their parameter had values in range 1.87–3.01. The presence of BH in the PS gel had a significant impact on the b* parameter, where the sample with juice (5%PS + 20%AJ) had more yellowness (11.80) than samples with BH (5%PS + 0.05%BH + 20%AJ and 5%PS + 0.10%BH + 20%AJ), b* parameter 9.67 and 9.43, respectively.

Corn starch gels (CS, WCS) had lower values of the L* parameter (60.51 and 70.52, respectively) (Table 7) than those in potato starches (PS, WPS) (70.72 and 76.07) (Table 6). In both samples, CS and WCS, we observed a statistical decrease in lightness (L*) parameters and also statistical differences between kissel with juice and fiber additions (5%CS + 20%AJ, 5%CS + 0.05%BH + 20%AJ, and 5%CS + 0.10%BH + 20%AJ). The buckwheat fiber contained in the samples caused the share of red color to increase with the increasing share of this additive, and this relationship was statistically significant. Thus, we observed the highest value obtained in the sample 5%CS + 0.10%BH + 20%AJ (2.86), and the lowest, of course, in the gel without additives (−1.82). In turn, parameter b* had the highest value in the kissel with juice (12.20), indicating that the yellow color was dominated and the addition of BH (0.05% and 0.10%) decreased this value with statistical significance.

### 3.5. Total Polyphenol Content of Kissel

The total content of polyphenols in the tested samples are presented in Table 8 and Table 9. Polyphenols were not determined in starch gels without additives because these gels contained only starches. On the other hand, both in gels based on potato starch and corn starch, the content of polyphenols resulted from the presence of apple juice because no statistically significant differences in the content of these compounds were observed in gels with buckwheat fiber compared to gels with apple juice alone.

The obtained starch-based desserts showed antioxidant capacity, which was determined using ABTS cation and DPPH radicals. As in the case of the total content of polyphenols, and in the study of the antioxidant capacity of desserts based on potato and corn starch, the main role of the additive with antioxidant properties was played by apple juice. The addition of buckwheat fiber caused a slight increase in these values, but it was not statistically significant.

## 4. Discussion

The quality of the final product is affected by many factors, such as the conditions of preparation, the way it is packaged, as well as storage. The parameters of the raw materials used in production and their mutual proportions also play an important role. According to the Niechlów. P.P.Z. S.A. [42], the water content of starch should not exceed 20%, which is related to the microbiological risk of the material and its rapid spoilage. The moisture content of all tested starches is within the indicated range. The buckwheat hulls used to enrich the kissel are a good source of dietary fiber, especially the insoluble fraction (62.06%). This fraction includes cellulose, hemicelluloses, and lignin. The content of the insoluble dietary fiber in different foodstuffs varies and is lower than in buckwheat hulls in apple fiber (48.7 g/100 g IDF) and tomato fiber (57.6 g/100 g IDF), but higher in oat fiber (73.6 g/100 g IDF) [43]. As a by-product, buckwheat hull is a promising raw material since it is a cheap and resourceful agricultural bioproduct.

The addition of juice to potato starch mixture caused a delay in the pasting onset as the T_0_ parameter was higher than in normal or waxy potato starch without additives (Table 2). Thus, the increase in the gelatinization temperature of potato starch was affected by the acidic environment caused by the apple juice in the kissel recipe. The measured pH of the juice was 3.45. According to Kaur et al. [44], the pasting temperature of acidified starches was higher than native starches. The lack of influence of dietary fiber addition on the T_0_ of starch pasting may be due to its low amount in the mixture. Indeed, as confirmed by our previous study, the addition of BH at a concentration of 0.2% (*w*/*w*) to a 4.8% potato starch solution increased T_0_ by 2.2 °C, and the effect was statistically significant [35]. Such a correlation was not observed for waxy potato starch pastes [35], as in the present work (Table 2). Thus, it can be assumed that dietary fiber at a sufficiently high concentration reduces leaching out of amylose. This is also confirmed by the results of another parameter—maximum viscosity. Amylose content in starch results in a higher maximum peak viscosity of the paste [45], that is why the normal potato starch samples reached a higher value of η_max_ than waxy starch (Figure 1 and Figure 2). Moreover, η_max_ is associated with a degree of granule swelling during heating, so the higher peak viscosity of starch indicates its higher water-holding capacity [46,47]. In the present research, the addition of apple juice to the potato starch mixture reduced the maximum viscosity of the pastes by almost 3.8 times. Furthermore, in this case, the effect of BH was statistically insignificant (Table 2). In the discussed systems among the two additives, the influence of apple juice proved to be dominant over that of the buckwheat hulls. According to Kaur et al. (2011) [44], the acid hydrolysis due to removing the amorphous regions of starch granules leads to the lowering of its viscosity.

Setback parameters were higher for normal potato starch systems than for the waxy starch (Table 2). The addition of apple juice caused an increase in setback parameters. The setback parameter calculated as the difference between the viscosity after cooling the paste to 50 °C and the minimum viscosity, allows us to determine the tendency of the starch to start the retrogradation process. The greater the difference between the aforementioned viscosities, the greater the value of the setback parameter, and, consequently, the tendency to retrograde is also stronger [48].

The normal potato starch with juice showed lower final viscosity values than the control sample (Table 2). This indicates that the final viscosity parameter (η_50 °C_) gelatinized the acidic potato starch system with juice which re-associated and formed a network structure slower than the control did. Moreover, it confirms that the final viscosity of the normal or waxy potato starch without additives forms more rigid gels than samples with juice and fiber additions [49]. The same effect was observed in the samples with waxy starch, where the presence of juice during gelatinization of the WPS reduced its final viscosity compared to the control sample [50].

The addition of natural fibers to starches influences their properties. Different fibers have been added to starches, such as those from wheat, cotton, oat, apple, pea, coir, vegetal, or barley-glucan [35,51,52,53,54]. In the present study, the buckwheat hulls and juice caused a decrease in T_0_ parameter and increased the final viscosity of waxy corn starch mixtures (Table 3). It should be noted, however, that for the other mixtures with normal potato starch and waxy potato and corn starch, a decrease in final viscosity was noted. Kaur et al. (2007) [55] studied the effect of aqueous HCl on the properties of wheat starch and found that the pasting temperature, peak viscosity, hot paste viscosity, and cold paste viscosity showed a decline with acid modification.

The hardness of fruit gels with buckwheat fiber was presented in Table 4 and Table 5. In the studies conducted, gels based on native starches were characterized by higher hardness. According to Krystyjan et al. (2022) [48], the hardness of starch gels increased with the storage time and the content of amylose. The low amylose content in starch (waxy forms) results in lower hardness of starch gels [56]. In this study, both the effect of pH and the addition of BH were found to be significant (Table 3 and Table 4). The effect of these two factors contributed to lowering the hardness of the obtained gels. A similar trend was observed in our previous research on the impact of BH on the textural properties of native potato starch gels [35]. However, it is worth noting that such changes were observed only at higher concentrations of polysaccharides (4.8% starch and 0.2% BH). At lower concentrations (3.8% starch and 0.2% BH), no differences were shown [35]. It is of interest to note that the pH (acidic condition) in all starch systems, except those with CS, affected the lowering of the hardness of the gels, and only the system with corn starch showed a different trend. This may be due to the difference in the size of granules between starches. Smaller granules (in CS) are more resistant to acid, and they also require higher temperatures for pasting.

Turbidimetric measurements are one of the methods for determining the retrogradation of starch gels, both short- and long-term. This method uses the phenomenon of gel turbidity during their aging [57]. Due to the fact that the amount of turbidity of the starch paste, immediately after its cooling, is influenced mainly by the amylose content, a short-term retrogradation is observed already at this time. Therefore, low concentrations of starch in the mixture should be used in order to observe this phenomenon in a given time. Studies using low concentrations of normal and waxy potato starch were conducted by Sikora et al. (2015) [58] and Krystyjan et al. (2016) [59]. Based on our previous experience, it was found that in order to compare the degree of turbidity of potato and corn starch gels, the color measurement method in the CIE L*a*b* system should be used, and the L* parameter will allow the observation of changes in the turbidity of gels with a concentration higher than 1%. An instrumental method of color determination is able to determine color differences between the tested samples in a very accurate and objective way. Moreover, color is one of the most important parameters during the organoleptic evaluation of a newly developed product. It also has a significant impact on the consumer’s decision when choosing food products [60]. The 5% concentration of starch in kissel allowed us to obtain samples of an appropriate consistency. The thickening base in the present systems was potato starch (normal and waxy) and corn starch (normal and waxy). Waxy forms of starch are appreciated in the design of various types of desserts because they are characterized by the appropriate consistency and hardness of the resulting gels. Native starch gels are white, but not transparent, while waxy starches are more transparent [61]. Low content of amylose in waxy starches is beneficial for a high transparency of the pastes [62]. Potato starch granules are larger in size than corn starch so that, as claimed Singh and Singh (2001) [56], such starches exhibit less gel turbidity, and thus are less susceptible to retrogradation.

The designed fruit gels (based on apple juice and with the addition of buckwheat fiber) contained phenolic compounds, which was confirmed by spectrophotometric analysis (Table 8 and Table 9). The addition of apple juice increased these parameters as apples are a rich source of flavonoids and phenolic acids. The main flavonoids in apples are quercetin and its glycosides, (−)-epicatechin, (+)-catechin, and phlorizin and its derivatives [63]. Phenolic acids are also present in apple flesh and skin (e.g., chlorogenic acid and others). The quantitative and qualitative profile of phenolic compounds found in apples differs depending on many factors (their variety, country of origin, etc.) [64]. Buckwheat hulls, in addition to their high fiber content, may be a valuable source of phenolic compounds [65] and have higher phenolic contents and antioxidant properties in comparison to buckwheat groats [66]. Some authors showed that the addition of ground buckwheat hulls at a level of 1–3% improved the nutritional value of meat products but also caused changes in the technological and sensory qualities (color and taste) [67].

## 5. Conclusions

The presence of apple juice and fiber in the blends had an impact on potato and corn starch pastes and gels. The addition of apple juice as well as dietary fiber affected the rheology and texture of the final product. When designing desserts with starch as a base, these are very important parameters that directly affect the consumer acceptance of the food. The presence of acidic juice in waxy corn starch pastes contributed to the reduction of the final viscosity of the tested systems. The addition of buckwheat hulls to kissel enriched the food product in fiber and phenolic compounds. Thus, the addition of buckwheat hulls and apple juice improved the nutritional value of the final products but also caused changes in the technological qualities (hardness and color). The results obtained open up possibilities for further research on the enrichment of food with nutritional health-promoting and functional components, as well as the use of raw by-products rich in micro- and macroelements, polyphenols, and fiber valuable to our health.

## Figures and Tables

**Table 1:** Composition of kissel.

Sample	Ingredient [%]							Sum
	PS	WPS	CS	WCS	AJ	BH	Water	
Normal potato starch								
5%PS + 20%AJ	5.00	–	–	–	20.00	–	75.00	100.00
5%PS + 0.05%BH + 20%AJ	5.00	–	–	–	20.00	0.05	74.95	100.00
5%PS + 0.10%BH + 20%AJ	5.00	–	–	–	20.00	0.10	74.90	100.00
Waxy potato starch								
5%WPS + 20%AJ	–	5.00	–	–	20.00	–	75.00	100.00
5%WPS + 0.05%BH+ 20%AJ	–	5.00	–	–	20.00	0.05	74.95	100.00
5%WPS + 0.10%BH+ 20%AJ	–	5.00	–	–	20.00	0.10	74.90	100.00
Normal corn starch								
5%CS + 20%AJ	5.00	–	–	–	20.00	–	75.00	100.00
5%CS + 0.05%BH + 20%AJ	5.00	–	–	–	20.00	0.05	74.95	100.00
5%CS + 0.10%BH + 20%AJ	5.00	–	–	–	20.00	0.10	74.90	100.00
Waxy corn starch								
5%WCS + 20%AJ	–	5.00	–	–	20.00	–	75.00	100.00
5%WCS + 0.05%BH + 20%AJ	–	5.00	–	–	20.00	0.05	74.95	100.00
5%WCS + 0.10%BH + 20%AJ	–	5.00	–	–	20.00	0.10	74.90	100.00

PS—potato starch; WPS—waxy potato starch; CS—corn starch, WCS—waxy corn starch; BH—buckwheat fiber; AJ—apple juice.

**Table 2:** Parameters of the pasting characteristics of potato starch gels and starch gels with buckwheat fiber.

Sample	T_0_ [°C]	η_max_ [BU]	T_ŋmax_ [°C]	η_95 °C_ [BU]	BD [BU]	SB [BU]	η_50 °C_ [BU]
5%PS (control)	61.2 ± 0.0 ^a^	1957.0 ± 10.0 ^b^	66.7 ± 0.1 ^a^	787.0 ± 2.0 ^b^	1325.0 ± 10.0 ^b^	70.5 ± 13.0 ^a^	706.0 ± 16.0 ^b^
5%PS + 20%AJ	70.1 ± 0.1 ^b^	507.0 ± 19.1 ^a^	81.4 ± 1.1 ^b^	477.5 ± 17.9 ^a^	66.5 ± 5.0 ^a^	209.5 ± 7.8 ^b^	649.5 ± 21.9 ^a^
5%PS + 0.05%BH + 20%AJ	70.1 ± 0.1 ^b^	517.0 ± 4.2 ^a^	81.1 ± 0.4 ^b^	486.0 ± 2.8 ^a^	70.5 ± 2.1 ^a^	211.5 ± 2.1 ^b^	658.0 ± 4.2 ^a^
5%PS + 0.10%BH + 20%AJ	70.1 ± 0.1 ^b^	518.5 ± 2.1 ^a^	80.7 ± 0.1 ^b^	488.5 ± 2.1 ^a^	70.5 ± 0.7 ^a^	209.5 ± 0.7 ^b^	657.5 ± 3.5 ^a^
Parameters marked with the same letter in the column do not differ significantly at a confidence level of α = 0.05.							
5%WPS (control)	67.6 ± 0.1 ^b^	1387.0 ± 0.2 ^b^	72.9 ± 0.0 ^a^	575.0 ± 5.1 ^b^	904.0 ± 8.8 ^a^	7.5 ± 1.0 ^a^	482.0 ± 11.0 ^b^
5%WPS + 20%AJ	70.1 ± 0.0 ^a^	1330.5 ± 19.1 ^a^	75.0 ± 0.1 ^b^	403.5 ± 9.2 ^a^	1080.0 ± 2.5 ^b^	34.5 ± 3.5 ^b^	216.0 ± 9.9 ^a^
5%WPS + 0.05%BH + 20%AJ	70.1 ± 0.1 ^a^	1328.5 ± 0.7 ^a^	75.0 ± 0.0 ^b^	407.5 ± 2.1 ^a^	1079.0 ± 2.1 ^b^	34.5 ± 0.7 ^b^	214.5 ± 2.1 ^a^
5%WPS + 0.10%BH + 20%AJ	70.0 ± 0.0 ^a^	1341.5 ± 0.7 ^a^	75.0 ± 0.1 ^b^	400.5 ± 7.8 ^a^	1094.5 ± 2.1 ^b^	32.5 ± 0.7 ^b^	214.5 ± 2.1 ^a^
Parameters marked with the same letter in the column do not differ significantly at a confidence level of α = 0.05.							

T_0_ (°C)—temperature of the beginning of pasting; η_max_ (BU)—maximum viscosity; T_η max_ (°C)—temperature at maximum viscosity; η_95 °C_ (BU)—viscosity at 95 °C; BD (BU)—breakdown; SB (BU)—setback; η_50 °C_ (BU)—viscosity after cooling to 50 °C; BU—Brabender Units; PS—potato starch; WPS—waxy potato starch; BH—buckwheat fiber; AJ—apple juice.

**Table 3:** Parameters of the pasting characteristics of corn starch gels and starch gels with buckwheat fiber.

Sample	T_0_ [°C]	η_max_ [BU]	T_ŋmax_ [°C]	η_95 °C_ [BU]	BD [BU]	SB [BU]	η_50 °C_ [BU]
5%CS (control)	86.0 ± 0.3 ^b^	110.0 ± 4.6 ^a^	94.2 ± 0.0 ^b^	110.0 ± 5.6 ^a^	6.0 ± 0.0 ^a^	68.0 ± 0.0 ^a^	190.0 ± 10.0 ^a^
5%CS + 20%AJ	84.2 ± 0.1 ^a^	175.5 ± 3.5 ^b^	92.4 ± 0.0 ^a^	169.0 ± 4.0 ^b^	40.0 ± 1.0 ^b^	85.0 ± 7.0 ^b^	220.5 ± 4.5 ^b^
5%CS + 0.05%BH + 20%AJ	84.2 ± 0.1 ^a^	177.5 ± 5.5 ^b^	92.5 ± 0.2 ^a^	170.5 ± 4.5 ^b^	41.0 ± 3.0 ^b^	75.0 ± 7.0 ^b^	211.5 ± 9.5 ^b^
5%CS + 0.10%BH + 20%AJ	84.1 ± 0.1 ^a^	181.5 ± 1.5 ^b^	92.4 ± 0.1 ^a^	174.0 ± 2.0 ^b^	42.0 ± 1.0 ^b^	86.0 ± 5.0 ^b^	225.5 ± 5.5 ^b^
Parameters marked with the same letter in the column do not differ significantly at a confidence level of α = 0.05.							
5%WCS (control)	69.2 ± 0.1 ^a^	466.0 ± 1.5 ^a^	74.6 ± 0.8 ^a^	188.0 ± 1.2 ^b^	306.0 ± 3.0 ^a^	35.5 ± 0.5 ^b^	196.0 ± 10.0 ^b^
5%WCS + 20%AJ	71.2 ± 0.1 ^b^	502.0 ± 1.4 ^b^	76.3 ± 0.1 ^b^	137.0 ± 2.8 ^a^	401.5 ± 0.7 ^b^	18.0 ± 0.0 ^b^	118.5 ± 0.7 ^a^
5%WCS + 0.05%BH + 20%AJ	71.4 ± 0.2 ^b^	493.5 ± 17.7 ^b^	76.3 ± 0.3 ^b^	133.5 ± 7.8 ^a^	399.0 ± 11.3 ^b^	15.5 ± 0.7 ^a^	110.0 ± 0.7 ^a^
5%WCS + 0.10%BH + 20%AJ	71.3 ± 0.1 ^b^	504.5 ± 9.2 ^b^	76.2 ± 0.1 ^b^	135.5 ± 2.1 ^a^	406.5 ± 7.8 ^b^	16.5 ± 0.7 ^ab^	114.5 ± 0.7 ^a^
Parameters marked with the same letter in the column do not differ significantly at a confidence level of α = 0.05.							

T_0_ (°C)—temperature of the beginning of pasting; η_max_ (BU)—maximum viscosity; T_ηmax_ (°C)—temperature at maximum viscosity; η_95 °C_ (BU)—viscosity at 95 °C; BD (BU)—breakdown; SB (BU)—setback; η_50 °C_ (BU)—viscosity after cooling to 50 °C; BU—Brabender Units; CS—corn starch; WCS—waxy corn starch; BH—buckwheat fiber; AJ—apple juice.

**Table 4:** Hardness of fruit gels with potato starch and buckwheat fiber.

Sample	Hardness of Samples [N]
5%PS (control)	0.044 ± 0.004 ^a^
5%PS + 20%AJ	0.330 ± 0.010 ^d^
5%PS + 0.05%BH + 20%AJ	0.180 ± 0.000 ^c^
5%PS + 0.10%BH + 20%AJ	0.100 ± 0.020 ^b^
Parameters marked with the same letter in the column do not differ significantly at a confidence level of α = 0.05	
5%WPS (control)	0.023 ± 0.003 ^d^
5%WPS + 20%AJ	0.020 ± 0.002 ^b^
5%WPS + 0.05%BH + 20%AJ	0.022 ± 0.001 ^c^
5%WPS + 0.10%BH + 20%AJ	0.018 ± 0.002 ^a^
Parameters marked with the same letter in the column do not differ significantly at a confidence level of α = 0.05	

PS—potato starch; WPS—waxy potato starch; BH—buckwheat fiber; AJ—apple juice.

**Table 5:** Hardness of fruit gels with corn starch and buckwheat fiber.

Sample	Hardness of Samples [N]
5%CS (control)	0.199 ± 0.016 ^a^
5%CS + 20%AJ	0.380 ± 0.010 ^b^
5%CS + 0.05%BH + 20%AJ	0.390 ± 0.020 ^b^
5%CS + 0.10%BH + 20%AJ	0.510 ± 0.040 ^c^
Parameters marked with the same letter in the column do not differ significantly at a confidence level of α = 0.05	
5%WCS (control)	0.027 ± 0.040 ^c^
5%WCS + 20%AJ	0.021 ± 0.001 ^b^
5%WCS + 0.05%BH + 20%AJ	0.019 ± 0.001 ^a^
5%WCS + 0.10%BH + 20%AJ	0.018 ± 0.002 ^a^
Parameters marked with the same letter in the column do not differ significantly at a confidence level of α = 0.05	

CS—corn starch; WCS—waxy corn starch; BH—buckwheat fiber; AJ—apple juice.

**Table 6:** Color parameters (L*, a*, and b*) of samples with normal and waxy potato starch, buckwheat fiber and juice.

Sample	Color Parameters		
	L*	a*	b*
5%PS (control)	70.72 ± 0.00 ^b^	−1.38 ± 0.01 ^a^	4.70 ± 0.01 ^a^
5%PS + 20%AJ	32.93 ± 0.78 ^a^	3.01 ± 1.12 ^b^	11.80 ± 1.32 ^c^
5%PS + 0.05%BH + 20%AJ	30.50 ± 0.97 ^a^	2.48 ± 0.39 ^b^	9.67 ± 1.07 ^b^
5%PS + 0.10%BH + 20%AJ	32.85 ± 1.30 ^a^	1.87 ± 0.40 ^b^	9.43 ± 1.12 ^b^
Parameters marked with the same letter in the column do not differ significantly at a confidence level of α = 0.05.			
5%WPS (control)	76.07 ± 0.17 ^d^	−1.48 ± 0.02 ^a^	4.14 ± 0.16 ^a^
5%WPS + 20%AJ	62.30 ± 0.08 ^c^	5.90 ± 0.03 ^b^	42.60 ± 0.04 ^c^
5%WPS + 0.05%BH + 20%AJ	58.20 ± 0.17 ^b^	6.10 ± 0.03 ^c^	37.70 ± 0.14 ^c^
5%WPS + 0.10%BH + 20%AJ	52.50 ± 0.23 ^a^	6.80 ± 0.03 ^d^	32.50 ± 0.26 ^b^
Parameters marked with the same letter in the column do not differ significantly at a confidence level of α = 0.05.			

PS—potato starch; WPS—waxy potato starch; BH—buckwheat fiber; AJ—apple juice.

**Table 7:** Color parameters (L*, a*, and b*) of samples with normal and waxy corn starch, buckwheat fiber and juice.

Sample	Color Parameters		
	L*	a*	b*
5%CS (control)	60.51 ± 0.06 ^d^	−1.82 ± 0.03 ^a^	−4.37 ± 0.12 ^a^
5%CS + 20%AJ	50.18 ± 0.14 ^c^	1.14 ± 0.12 ^b^	12.20 ± 0.27 ^d^
5%CS + 0.05%BH + 20%AJ	46.85 ± 0.28 ^b^	2.14 ± 0.24 ^c^	11.27 ± 0.38 ^c^
5%CS + 0.10%BH + 20%AJ	44.25 ± 0.13 ^a^	2.86 ± 0.02 ^d^	10.51 ± 0.30 ^b^
Parameters marked with the same letter in the column do not differ significantly at a confidence level of α = 0.05.			
5%WCS (control)	70.52 ± 0.09 ^d^	−0.95 ± 0.03 ^a^	7.45 ± 0.02 ^a^
5%WCS + 20%AJ	56.00 ± 0.60 ^c^	5.65 ± 0.12 ^b^	33.91 ± 0.68 ^d^
5%WCS + 0.05%BH + 20%AJ	51.70 ± 1.15 ^b^	6.81 ± 0.22 ^c^	30.59 ± 1.31 ^c^
5%WCS + 0.10%BH + 20%AJ	47.20 ± 0.15 ^a^	7.71 ± 0.03 ^d^	26.43 ± 0.17 ^b^
Parameters marked with the same letter in the column do not differ significantly at a confidence level of α = 0.05.			

CS—corn starch; WCS—waxy corn starch; BH—buckwheat fiber; AJ—apple juice.

**Table 8:** Total polyphenol content in samples with normal and waxy potato starch, buckwheat fiber and juice.

Sample	Total Polyphenols Content [mg GAE/100 g]	ABTS mM TE/100 g	DPPH mM TE/100 g
5%PS (control)	0.00 ± 0.00 ^b^	0.00 ± 0.00 ^b^	0.0000 ± 0.0000 ^b^
5%PS + 20%AJ	129.36 ± 4.08 ^a^	0.13 ± 0.01 ^a^	0.0173 ± 0.0007 ^a^
5%PS + 0.05%BH + 20%AJ	133.02 ± 3.80 ^a^	0.15 ± 0.01 ^a^	0.0174 ± 0.0005 ^a^
5%PS + 0.10%BH + 20%AJ	134.35 ± 1.40 ^a^	0.15 ± 0.01 ^a^	0.0176 ± 0.0004 ^a^
Parameters marked with the same letter in the column do not differ significantly at a confidence level of α = 0.05			
5%WPS (control)	0.00 ± 0.00 ^b^	0.00 ± 0.00 ^b^	0.0000 ± 0.0000 ^b^
5%WPS + 20%AJ	57.6 ± 0.47 ^a^	0.12 ± 0.01 ^a^	0.0172 ± 0.0004 ^a^
5%WPS + 0.05%BH + 20%AJ	53.6 ± 0.26 ^a^	0.13 ± 0.01 ^a^	0.0173 ± 0.0005 ^a^
5%WPS + 0.10%BH + 20%AJ	51.3 ± 0.34 ^a^	0.14 ± 0.01 ^a^	0.0173 ± 0.0004 ^a^
Parameters marked with the same letter in the column do not differ significantly at a confidence level of α = 0.05			

PS—potato starch; WPS—waxy potato starch; BH—buckwheat fiber; AJ—apple juice.

**Table 9:** Total polyphenol content in samples with normal and waxy corn starch, buckwheat fiber and juice.

Sample	Total polyphenol Content [mg GAE/100 g]	ABTS mM TE/100 g	DPPH mM TE/100 g
5%CS (control)	0.00 ± 0.00 ^b^	0.00 ± 0.00 ^b^	0.0000 ± 0.0000 ^b^
5%CS + 20%AJ	57.74 ± 4.65 ^a^	0.13 ± 0.01 ^a^	0.0170 ± 0.0004 ^a^
5%CS + 0.05%BH + 20%AJ	53.63 ± 2.48 ^a^	0.14 ± 0.01 ^a^	0.0173 ± 0.0004 ^a^
5%CS + 0.10%BH + 20%AJ	51.28 ± 3.31 ^a^	0.15 ± 0.01 ^a^	0.0173 ± 0.0004 ^a^
Parameters marked with the same letter in the column do not differ significantly at a confidence level of α = 0.05.			
5%WCS (control)	0.00 ± 0.00 ^b^	0.00 ± 0.00 ^b^	0.0000 ± 0.0000 ^b^
5%WCS + 20%AJ	41.67 ± 1.59 ^a^	0.12 ± 0.01 ^a^	0.0171 ± 0.0004 ^a^
5%WCS + 0.05%BH + 20%AJ	47.04 ± 6.91 ^a^	0.13 ± 0.01 ^a^	0.0172 ± 0.0004 ^a^
5%WCS + 0.10%BH + 20%AJ	50.68 ± 3.77 ^a^	0.14 ± 0.01 ^a^	0.0173 ± 0.0004 ^a^
Parameters marked with the same letter in the column do not differ significantly at a confidence level of α = 0.05.			

CS—corn starch; WCS—waxy corn starch; BH—buckwheat fiber; AJ—apple juice.

## Data Availability

The data that support the findings of this study are available from the corresponding author upon reasonable request.
